# Phaco-UCP; combined phacoemulsification and ultrasound ciliary plasty versus phacoemulsification alone for management of coexisting cataract and open angle glaucoma: a randomized clinical trial

**DOI:** 10.1186/s12886-021-01818-5

**Published:** 2021-01-21

**Authors:** Magda A. Torky, Yousef A. Alzafiri, Ameera G. Abdelhameed, Eman A. Awad

**Affiliations:** 1 Department of Ophthalmology, Mansoura Ophthalmic Center, Faculty of medicine, Mansoura University, Al-Gomhoria Street, Mansoura, 35516 Egypt; 2 Department of Ophthalmology, Dar Al Shifa hospital, 30000 Hawally City, Kuwait

**Keywords:** Phaco-UCP, Ultrasound ciliary plasty, Phacoemulsification, OAG

## Abstract

**Background:**

Various surgical techniques have been described, to be combined with cataract surgery in glaucoma patients, aiming for an additional reduction of intraocular pressure (IOP), hence minimizing the burden of anti-glaucoma medication (AGM). Ultrasound ciliary plasty (UCP) is a recent microinvasive glaucoma surgery (MIGS) recommended for primary and refractory glaucoma. This study was conducted to evaluate the safety and efficacy of a new technique; combined phacoemulsification and ultrasound ciliary plasty (Phaco-UCP) as a primary surgical treatment for coexisting cataract and open angle glaucoma.

**Methods:**

A randomized clinical trial, including 61 eyes of 61 patients with visually significant cataract and open angle glaucoma, randomized to either Phaco-UCP (study group; 31 eyes) or phacoemulsification alone (Phaco-alone) (control group; 30 eyes). Primary outcomes included reduction in IOP and/or the number of AGM. Secondary outcomes included visual acuity improvement and complications. Qualified Success was defined as an IOP reduction ≥ 20% from baseline value, with an IOP 6–21 mmHg, with no additional AGM or glaucoma surgery. Failure was defined as either < 20% IOP reduction, despite AGM use, the need of glaucoma surgeries or serious complications.

**Results:**

At 18 months postoperatively, Phaco-UCP group had a median IOP reduction of 7 mmHg (Q1, Q3 = 3, 10) compared to 2 mmHg (Q1, Q3 = 2, 3) in Phaco-alone group (*P* < 0.001). Phaco-UCP group had significantly higher success rate at all time points reaching 67.7% at the last follow-up versus 16.7% only in Phaco-alone group (*P*< 0.001). The median number of AGM significantly decreased from [3 (Q1, Q3 = 2, 4), 3 (Q1, Q3 = 2,3)] respectively, (*P* =0.3)] at baseline to [1 (Q1,Q3 = 1, 2), 2 (Q1,Q3 = 2, 2)] respectively, (*P* < 0.001)] at 18 months postoperatively. No serious intraoperative or postoperative complications were encountered in either group.

**Conclusion:**

Phaco-UCP is a simple, safe and effective procedure for management of coexisting cataract and open angle glaucoma.

**Trial registration:**

ClinicalTrials.gov identifier, NCT04430647; retrospectively registered. June 12, 2020.

## Background

Cataract and glaucoma are leading causes of blindness all over the world (51 and 8%, respectively), that frequently coexist in the same eye [1]. Cataract surgery has been found to decrease the intraocular pressure (IOP) in both normal and glaucomatous eyes, by an average of 1.5–4 mmHg [2, 3]. However, this reduction is influenced by many factors including preoperative IOP, angle configuration and type of glaucoma [4]. IOP reduction is more with higher preoperative IOP [2] and in eyes with closed angles more than those with open angles [4]. In eyes with moderate to advanced open angle glaucoma, relying on phacoemulsification alone to reduce IOP may not be sufficient. In addition, the effect of IOP reduction after phacoemulsification alone is known to regress over time [5]. Moreover, IOP spikes after phacoemulsification is one of the feared complications in moderate and advanced stages of glaucoma [6].

Many glaucoma surgeons recommend a combined procedure for patients with significant cataract and glaucoma requiring urgent drainage surgery [7]. Phaco-trabeculectomy (Phaco-trab) has been traditionally shown to be effective, but might be associated with significant complications, the commonest of which are hypotony, hyphema, and shallow anterior chamber [8]. Recently, various microinvasive glaucoma surgeries (MIGS) have been described, to be combined with cataract surgery, aiming for an additional reduction of IOP and hence, decreasing the burden of anti-glaucoma medication (AGM) [9].

Ultrasound ciliary plasty (UCP) is a recent non-incisional technique recommended for primary and refractory glaucoma [10, 11]. It involves inducing selective coagulation of the ciliary epithelium using the high intensity focused ultrasound technology (HIFU) [10]. It has the advantages of being easy, one-step, highly reproducible, and more precise treatment [12].

This study was carried out to evaluate the safety and efficacy of combined phacoemulsification and Ultrasound ciliary plasty (Phaco-UCP) as a first-line surgical treatment for coexisting cataract and open angle glaucoma, compared to phacoemulsification alone (Phaco-alone). To our knowledge, this is the first report of the results of combined Phaco-UCP.

## Methods

### Study design

This was a randomized clinical trial, conducted at ophthalmology department of Dar Al Shifa hospital, Kuwait during the period from September 2018 through March 2020 after local “Institutional Review Board” approval. A written informed consent was obtained from each patient after explanation of the study nature. The study protocol was adherent to the tenets of the Declaration of Helsinki and to CONSORT guidelines for reporting clinical trials. The study was retrospectively registered on www.clinicaltrials.gov (NCT04430647) available at https://www.clinicaltrials.gov/ct2/show/NCT04430647?cond=NCT04430647&draw=2&rank=1.

### Sample size

Sample size was calculated using Epi Info™ software (CDC, version 7.2.3.0). The significance level (α) and the statistical power were set at 0.05 and 0.80 respectively. To our knowledge, no previous study compared Phaco-UCP and Phacoemulsification alone. Therefore, the effect size was calculated based on the difference in proportions of successful reduction of IOP, reported in a previous study comparing combined phacoemulsification and Endoscopic cyclophotocoagulation (Phaco-ECP) and phacoemulsification alone [13], (7.5% in control group and 37.5% in the study group at 6 months postoperative). We assumed that Phaco-UCP can achieve the same success rate of Phaco-ECP. Using Fleiss method and assuming a 1:1 ratio of groups, the total calculated sample size was 60 (30 per group). Ten percent expected attrition was added to the sample size to account for loss to follow-up, so the final sample size was 66 patients (33 per group).

### Inclusion/exclusion criteria

Patients with primary open angle glaucoma (POAG) or pseudoexfoliation glaucoma with coexisting visually significant cataract that required phacoemulsification were included. Visually significant cataract was defined, according to LOCS III criteria as: nuclear cataract ≥ 3/6.9, cortical cataract ≥3/5.9 or posterior subcapsular cataract ≥ 2/5.9 [14]. POAG was defined as optic neuropathy with typical glaucomatous optic disc cupping and visual field changes together with an open angle (Shaffer grade 3 or 4). Pseudoexfoliation glaucoma was diagnosed if there was exfoliation material adherent to the lens surface or to the pupillary margin, wide-open angle with dense pigmentation, Sampaolesi’s line, and an optic neuropathy with matching visual field loss. Glaucoma severity was classified based on visual field mean deviation (MD) into mild (MD better than − 6 dB), moderate (MD − 6 dB or worse but better than − 12 dB) and advanced (MD is − 12 dB or worse) [15]. Only eyes with mild and moderate glaucoma were included. For patients who had both eyes operated, only one eye was included in the study. Patients with any other type of glaucoma; including narrow angle glaucoma, neovascular, uveitic or angle recession glaucoma, patients with advanced glaucoma and patients with history of glaucoma surgery, intraocular surgery, laser trabeculoplasty, laser refractive surgery or any ocular diseases that would affect safety or interfere with the procedure were all excluded. We also excluded patients who declined to participate as well as those with incomplete follow-up or missing data.

### Preoperative evaluation

Preoperative evaluation included manifest refraction, corrected distance visual acuity (CDVA) measurement, slit lamp biomicroscopy, gonioscopy, indirect ophthalmoscopy, measurement of IOP with Goldmann applanation tonometry (average of 3 readings taken). Corneal diameter (White-to-white) and axial length measurement were done using IOL Master 500 (Carl Zeiss Meditec AG., Germany). Ultrasound pachymetry with Tomey SP-100 (Tomey Corp. Nagoya, Japan) and visual fields using Humphrey Field Analyzer (24–2, SITA, standard program Carl Zeiss Meditec AG., Germany) were also performed.

### Randomization and masking

Patients were assigned by simple coin flip randomization for either combined phacoemulsification and ultrasound ciliary plasty (Phaco-UCP; the study group), or phacoemulsification alone (Phaco-alone; the control group). The random allocation sequence was generated by one of the authors (EAA). Allocation assignments were sealed in opaque envelopes labelled only with study identification numbers. Patients were enrolled and assigned to intervention by the same author (YAA). Patients were not blinded to the intervention, as they had to sign a written informed consent. All preoperative and postoperative assessments were performed by the same author (MAT), who was masked to group allocation.

### Surgical technique

All surgeries were performed by the same surgeon (YAA).

### Phaco-UCP

Under peribulbar anesthesia, using 2% lidocaine (Xylocaine 2%, AstraZeneca, Bangalore, India), UCP was performed first, followed by phacoemulsification. UCP was performed using the same technique described before [16]. For all treatments, 2nd generation probe was used (EyeOP1, Eye Tech care; France) with the same parameters: operating frequency was 21 MHz; number of sectors activated was 6; Acoustic power was 2.45 W; duration of each shot was 8 s; and the time between shots was 20s. Using this protocol, no more than 3 min would be added to the Phaco-time. The probe diameter (11, 12 or 13 mm) was determined according to the eye’s biometric readings. The coupling cone was centered on the eye and kept in place with low vacuum suction, followed by introduction of the treatment probe inside the cone, then activation of the transducers by constantly pressing the foot switch. Once UCP treatment was finished, phacoemulsification was commenced.

### Phacoemulsification

A standard phacoemulsification was performed with 2.2 mm clear corneal incision, continuous curvilinear capsulorhexis, phacoemulsification and implantation of foldable acrylic intraocular lens (AcrySof® IQ SN60WF monofocal; Alcon Laboratories Inc., Fort Worth, TX, USA) in the capsular bag. Irrigation-aspiration was performed for at least 30 s to remove any viscoelastic from the anterior chamber. Reformation of the anterior chamber was done with balanced saline solution (BSS), followed by hydration of the corneal wound and side port. Intracameral cefuroxime (AproKam®) and subconjunctival dexamethasone injection were used at the completion of surgery.

### Postoperative management

Follow-ups were scheduled at 1 day, 1 week, 1 month, 3 months, 6 months, 12 months and 18 months postoperatively. Patients were treated with moxifloxacin 0.05% eye drops six times daily for 1 month and prednisolone acetate 1% eye drops six times daily for 1 week followed by gradual withdrawal over 4 weeks. Preoperative anti-glaucoma medications were continued during the first postoperative month, and then gradually decreased at each subsequent visit if the IOP was maintained at the target level. They were added only if IOP exceeded 21 mmHg or when they were needed to treat visual field or optic nerve changes.

### Outcome measures

Primary outcome measures included reduction in IOP and/or the number of AGM. Secondary outcome measures included CDVA improvement, intraoperative and postoperative complications.

#### Success and failure rates

Qualified Success was defined as an IOP reduction of at least 20% from baseline value, with an IOP that is between 6 and 21 mmHg, without the need for additional AGM or glaucoma surgery [13, 17]. Failure was defined as either < 20% IOP reduction form baseline value, despite the use of antiglaucoma medications, the need of other glaucoma surgeries or the development of any serious complications. By serious complications, we mean hyphema or vitreous hemorrhage necessitating surgical intervention, choroidal hemorrhage, chronic uveitis, endophthalmitis, hypotony (IOP ≤ 5 mmHg), phthisis, intraocular lens dislocation, and retinal detachment.

### Statistical analysis

Data were tabulated and statistically analyzed using the Statistical Package for Social Sciences (SPSS) version 25. Qualitative data were described as numbers and percentages. While quantitative data were described as means (± SD) or medians (first and third quartiles: Q1, Q3). Shapiro-Wilk test was used to test normality. Between-groups comparison was done using Student t-test for normally distributed data like age and CDVA and Mann-Whitney U test for non-normally distributed variables. Within-group comparisons were done using Wilcoxon signed rank test and Freidman test. “*P* value ≤ 0.05” was considered to be statistically significant.

## Results

### Patients’ characteristics

**Fig. 1:**
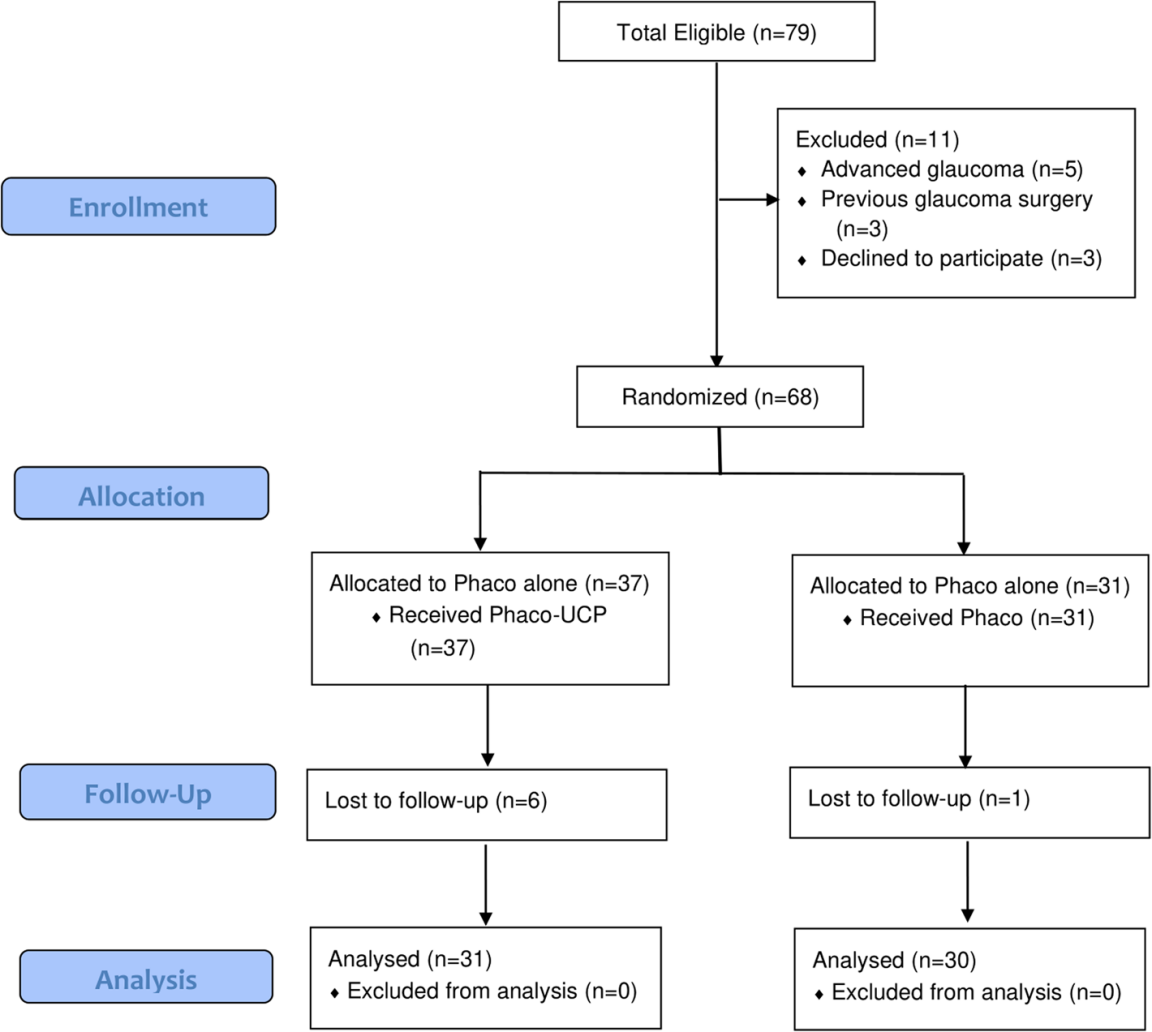
CONSORT Flowchart of study participants

A total of 61 eyes of 61 patients were randomly distributed into two groups: the study group (Phaco-UCP), including 31 eyes and the control group (Phaco-alone), including 30 eyes (Fig. 1). No statistically significant differences were found between the two groups regarding demographic and baseline clinical characteristics (Table 1).

**Table 1:** Demographic and baseline clinical characteristics of the study and control groups

Parameter	Study group (***n*** = 31)	Control group (***n*** = 30)	P	Test of significance
**Age; years** (mean ±SD)	59.1 ± 7.6	58.2 ± 12.6	0.7	Student t-test
**Gender** [no. (%)]			0.7	*x*^2^: Chi-square
Male	18 (58.1%)	16 (53.3%)		
Female	13 (41.9%)	14 (46.7%)		
**Type of glaucoma** [no. (%)]			0.7	*x*^2^: Chi-square
POAG	22 (71.0%)	20 (66.7%)		
PEX glaucoma	9 (29.0%)	10 (33.3%)		
**Number of AGM** median (Q1, Q3)	3 (2, 4)	3 (2, 3)	0.3	Mann-Whitney U
**Baseline IOP** (mmHg) Median (Q1, Q3)	24 (22, 28)	24 (22, 25)	0.3	Mann-Whitney U
**LogMAR (CDVA)**	0.89 ± 0.68	0.79 ±0.46	0.3	Student t-test
**Visual field MD (dB)**	- 8.2 ± 2.4 (−0.95 to −11.54)	- 8.1 ± 1.8 (− 2.87 to – 11.33)	0.9	Student t-test

*SD* Standard deviation, *POAG* Primary open angle glaucoma, *PEX* Pseudoexfoliation, *IOP* Intraocular pressure, *AGM* Anti-glaucoma medication, *CDVA* Corrected Distance Visual Acuity, *MD* Mean deviation

### Efficacy

#### IOP outcome

Table 2 compares the preoperative and postoperative IOP between the two groups. Both groups had a significantly lower IOP postoperatively. A significantly greater IOP reduction and percentage IOP reduction were observed in the study group compared to the control group at all-time points (Tables 3 and 4 respectively).

**Table 2:** Between-group and within-group comparison of preoperative and postoperative IOP

Timepoints	IOP (mmHg); Median (Q1, Q3)		
	Study group (***n***=31)	Control group (***n***=30)	***P 1***
**Baseline (preoperative)**	24 (22, 28)	24 (22, 25)	0.3
**1 day postoperative**	8 (6, 9)	10 (9, 12)	<0.001
**1 Week**	10 (8,11)	13 (11, 18)	<0.001
**1 Month**	13 (12,15)	17 (14, 22)	<0.001
**3 Months**	16 (14,20)	22 (17, 24)	<0.001
**6 Months**	18 (14, 19)	22 (16, 23)	<0.001
**12 Months**	16 (13, 19)	21 (17, 23)	<0.001
**18 Months**	17 (15, 19)	21 (18, 23)	<0.001
***P2***	<0.001	<0.001	
***P3***	<0.001	<0.001	

*IOP* Intraocular pressure*, Q1* 1st quartile*, Q3* 3rd quartile

***P 1:*** *Mann-Whitney U test between the study and control groups at each time point*

***P 2:*** *Freidman test between median IOP at baseline and at different time points postoperative within each group*

***P 3:*** *Wilcoxon Signed Ranks test between median IOP at baseline and at 18th month postoperative within each group*

**Table 3:** Between-group comparison of IOP reduction at different timepoints

Timepoints	IOP reduction (mmHg); Median (Q1, Q3)		
	Study group (***n***=31)	Control group (***n***=30)	P *
**1 day postoperative**	15 (15,21)	12 (11, 13)	<0.001
**1 Week**	13 (13, 15)	9 (5, 10)	<0.001
**1 Month**	11 (8,13)	5 (3, 7)	<0.001
**3 Months**	7 (4, 12)	2 (0, 4)	<0.001
**6 Months**	7 (4,10)	2 (1, 4)	<0.001
**12 Months**	8 (3, 11)	2 (1, 3)	<0.001
**18 Months**	7 (3,10)	2 (2, 3)	<0.001

*IOP* Intraocular pressure*, Q1* 1st quartile*, Q3* 3rd quartile

* Mann-Whitney U test

**Table 4:** Between-group comparison of percentage IOP reduction at different timepoints

Timepoints	Percentage IOP reduction (%); Median (Q1, Q3)		
	Study group (n=31)	Control group (n=30)	P *
**1 day postoperative**	67 (65, 71)	54 (50, 59)	<0.001
**1 Week**	58 (57, 62)	42 (22, 48)	<0.001
**1 Month**	48 (38, 50)	22 (12, 32)	<0.001
**3 Months**	30 (17, 47)	9 (0, 17)	<0.001
**6 Months**	29 (17, 42)	8 (4, 20)	<0.001
**12 Months**	36 (15, 43)	9 (6, 15)	<0.001
**18 Months**	32 (14, 41)	9 (8, 18)	<0.001

*IOP* Intraocular pressure; *Q1* 1st quartile, *Q3 3rd quartile*

* Mann-Whitney U test

**Fig. 2:**
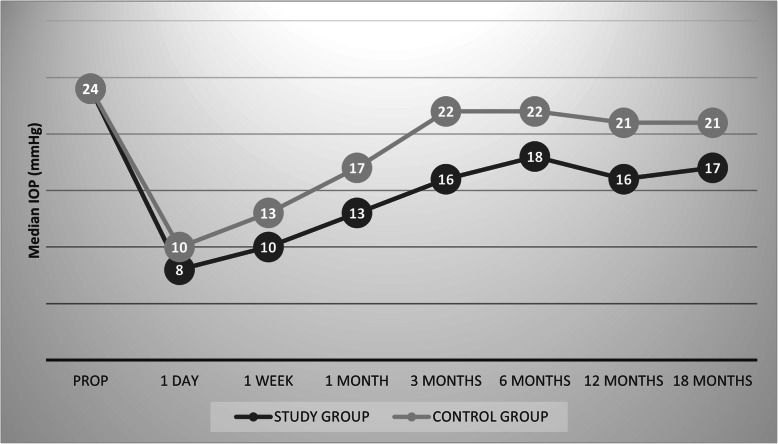
Changes in the median IOP (mmHg) in both groups at different time points

**Fig. 3:**
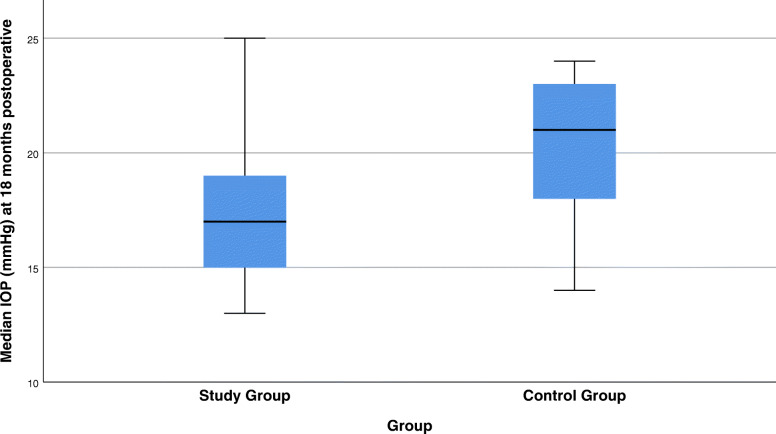
Postoperative median IOP (mmHg) in both groups at the 18th month follow-up visit

Figure 2 shows comparison of median IOP in both groups at different time points, while Fig. 3 shows boxplot for the median IOP at 18 months post-operative in both groups. The study group had a significantly higher success rate compared to the control group at all-time points, reaching 67.7% at the last follow up in the study group versus 16.7% only in the control group (Table 5).

**Table 5:** Between-group comparison of qualified success at different timepoints

Time	Qualified success (No, %)		P*
	Study group	Control group	
1st day	31 (100%)	30 (100%)	
1st Week	31 (100%)	26 (86.7%)	0.05
1st Month	29 (93.5%)	16 (53.3%)	<0.001
3rd Month	21 (67.7%)	7 (23.3%)	0.001
6th Month	21 (67.7%)	8 (26.7%)	0.002
12th Month	22 (71%)	6 (20%)	<0.001
18th Month	21 (67.7%)	5 (16.7%)	<0.001

*Chi-square test

#### Antiglaucoma medication outcome

Both groups had statistically significant reduction in the number of AGM at 18 months postoperatively (*p*< 0.001), with a significantly greater reduction in the study group compared to the control group [1 (Q1, Q3 = 1, 2), 2 (Q1, Q3 = 2, 2) respectively, (*P* < 0.001)] (Table 6).

**Table 6:** Between-group and within-group comparison of preoperative and postoperative number of antiglaucoma medication used

	Number of AGM Median (Q1, Q3)		P*
	Study group (n=31)	Control group (n=30)	
**Preoperative**	3 (2, 4)	3 (2, 3)	0.3
**18 months Postoperative**	1 (1,2)	2 (2, 2)	<0.001
P**	<0.001	<0.001	

*AGM* Antiglaucoma medication, *Q1* 1st quartile, *Q3 3rd quartile*

* Mann-Whitney

** Wilcoxon Signed Ranks test

#### Visual acuity outcome

Both groups had significant improvement in the mean LogMAR CDVA from a baseline of 0.89 ± 0.68 and 0.79 ±0.46 in the study and control group respectively to 0.25 ± 0.26 (*P* = 0.023) and 0.29 ± 0.36 (*P* = 0.014) respectively at 18 months postoperative. No patients in either groups lost CDVA at 18 months compared to baseline.

#### Safety and complications

All patients of the study group tolerated the procedure well with no serious intraoperative or postoperative complications (Table 7). The most frequent postoperative complication was anterior chamber flare, which occurred in all eyes and was treated by increasing the frequency of topical steroids with the help of Non-Steroidal Anti-inflammatory (NSAIDs) eye drops and resolved completely over 3–4 weeks. Three eyes (9.6%) developed fibrinous reaction, which resolved completely over 3–4 weeks with the use of the same regimen.

**Table 7:** Postoperative complications in both groups

Complications	Number of patients (%)		
	Study group (n = 31)	Control group (n = 30)	P*
Anterior chamber flare	31 (100%)	3 (10%)	0.014
Fibrin in anterior chamber	3 (9.6%)	3 (10%)	0.7
Mydriasis	3 (9.6%)	None (0%)	0.005
Macular oedema	4 (12.9%)	3 (10%)	0.6
Superficial Punctate keratitis	5 (16.1%)	None (0%)	0.001
Hyphema	None (0%)	1 (3.3%)	0.08

* Chi-square test

Mydriasis occurred in 3 eyes (9.6%) and resolved spontaneously after 6 months. Clinically significant macular oedema (CME) developed in four eyes (12.9%). However, it was transient and resolved after one month with NSAIDs drops four times a day, without affecting the final CDVA. Five eyes (16.1%) developed superficial punctate keratitis that resolved spontaneously in few days. Neither Hypotony (IOP ≤5 mmHg) nor IOP spikes (IOP > baseline IOP + 10 mmHg in the first 7 days) were encountered in any of the eyes. Finally, none of the eyes had choroidal detachments or phthisis.

In the control group, 3 patients (10%) had anterior chamber inflammation, 3 patients (3.3%) developed cystoid macular oedema, and one patient (3.3%) developed hyphema. All these complications resolved by appropriate treatment within 4 weeks of surgery.

## Discussion

Ultrasound ciliary plasty (UCP) is a noninvasive IOP-lowering technique that uses high-intensity focused ultrasound (HIFU) to achieve selective coagulation of the ciliary body, with a more predictable and controlled IOP reduction than traditional cyclodestructive procedures [10, 18]. Although its main IOP-lowering mechanism is reduction in aqueous humor inflow following ciliary epithelial thermal coagulation, an increase in suprachoroidal and transscleral aqueous humor outflow has also been reported [10, 19, 20]. Therefore, It has been used by many glaucoma surgeons for different types of glaucoma [10–12, 21, 22].

In our previous study [16], we reported the safety and efficacy of UCP as an initial treatment for primary and secondary open angle glaucoma. In the current study, we report the outcomes of a new combined procedure (Phaco-UCP) as a primary surgical treatment for coexisting open angle glaucoma and visually significant cataract, compared to phacoemulsification alone (Phaco-alone). We found that combining UCP with phacoemulsification resulted in significantly greater reduction of IOP and number of AGM than phacoemulsification alone, without jeopardizing the final visual acuity. To our knowledge, this is the first report of Phaco-UCP, so we will compare its results to those of combined phacoemulsification and endoscopic cyclophotocoagulation (Phaco-ECP), a well-known cyclophotocoagulation procedure, which provides selective destruction of ciliary body epithelium with less tissue disruption [23].

Francis et al. [13] reported that after a follow up period of 3 years, Phaco-ECP resulted in lower IOP and a greater reduction in AGM than Phaco-alone at all time points. However, they reported lower percentage IOP reduction, and lower success rates in both the study and control groups (percentage IOP reduction at 2 years 10.1 ± 17.1% and 0.8 ± 12.0% respectively and success rates at 2 years 13.8 and 3.8% respectively) than reported in the current study, most probably due to different study population. Their study included medically controlled POAG patients with a lower baseline IOP (18.1 ± 3.0 mmHg in both groups) and fewer number of AGM used preoperatively (1.5 ± 0.8, 2.4 ± 1.0 in the study and control groups respectively).

In a retrospective study by Pérez Bartolomé et al. [24], comparing Phaco-ECP to Phaco-alone in patients with POAG, success rate after one year for Phaco-ECP (69.6%) was similar to that reported in the current study (71%), yet with a lower percentage of IOP reduction (21.5%) compared to the current study (36%). Their Phaco-ECP group included patients with uncontrolled glaucoma, with previous failed surgery and 3 or more AGM. Interestingly, their Phaco-alone group had a comparable IOP reduction (1.9 ± 3.6 mmHg) and percentage IOP reduction (9.9 ± 7.5%) to our control group, however, with a higher success rate (40%). This might be due to the fact that patients in their Phaco-alone group had medically controlled early POAG with a lower baseline IOP (18.4 ± 3.7 mmHg), with one or two preoperative medications only.

Regarding safety, the less invasive nature of UCP made it possible to avoid the serious complications of traditional filtering surgery and implant-related complications of MIGS. The most common postoperative complication of Phaco-UCP encountered in our study was the anterior chamber reaction in the form of aqueous flare, seen in all treated eyes, starting at day one post-operatively and resolving over 3–4 weeks with intensive frequent topical steroid therapy and NSAIDs. Previous studies have reported significant increase of anterior chamber flare on the first day after UCP, followed by a gradual decrease, to recover to preoperative levels by 3 months postoperatively [22, 25]. However, the increased flare values after UCP were lower than those recorded after traditional cyclophotocoagulation [26]. Intraocular inflammation after UCP has been attributed to direct damage of ciliary epithelium, which is the key component of blood-aqueous barrier. Similarly, its resolution is proposed to follow the gradual recovery of blood-aqueous barrier, hence UCP was considered safe [22]. Moreover, the intraocular inflammation has been suggested to play a role in IOP reduction after cyclodestructive procedures [27, 28], due to release of some inflammatory mediators like prostaglandins, which enhances the uveoscleral aqueous outflow [29].

We reported a similar incidence of postoperative transient macular oedema for Phaco-UCP (12.9%) and Phaco-alone (10%), (*P* = 0.7). Hugo et al. [25] also reported a similar incidence (13%) after UCP alone. Mydriasis occurred in 3 eyes (9.6%) after Phaco-UCP and disappeared at the 6th month postoperative. Many authors have noticed changes in pupil shape and dynamics after UCP, both in phakic and pseudophakic eyes. Though a consensus has not been reached concerning the mechanism, this has been reported to be temporary and to resolve spontaneously after variable duration [16, 30–32].

In our hands, no serious complications occurred after Phaco-UCP. We observed that postoperative complications of combined Phaco and UCP were similar to those of UCP alone [10, 12, 21, 25]. For patients with coexistent open angle glaucoma and visually significant cataract, this combined procedure seems to be a better choice than phacoemulsification alone. Moreover, it may offer many advantages than the traditional phaco-trabeculectomy procedure. The easier technique of UCP allows for a shorter operation time and of course less postoperative complications. In addition, Phaco-UCP needs fewer postoperative follow-ups and additional interventions (including subconjunctival injection of 5-FU and laser suture lysis). Phaco-UCP might provide a temporary control of IOP in patients anticipating a future filtering surgery, until the ocular condition has improved enough to allow surgery. The option of repeated treatment with this non-invasive procedure (UCP) makes it feasible, effective and safe option in glaucoma management.

One of the limitations of our study is applying a simple randomization method, in the setting of a small sample size, which might yield unequal number of patients in each group, with different baseline characteristics, resulting in unreliable interpretation of results. However, we had nearly equal, matching groups regarding baseline characteristics. Another limitation is the short duration of follow-up (18 months), probably not reflecting the long-term outcome of the procedure. Future studies with larger samples, longer follow-up periods and stronger randomization techniques would be valuable to achieve more robust conclusion about the long-term efficacy of Phaco-UCP. Finally, this study was carried out on Asian population only. Racial differences may influence pigmentation of ocular structures including the ciliary epithelium, consequently enhancing or compromising Phaco-UCP effect [33]. The efficacy and safety of Phaco-UCP procedure in other ethnicities should be further evaluated.

## Conclusion

This is the first report of the novel technique of Phaco-UCP. The favorable findings of this study suggest that combining UCP to phacoemulsification does not compromise the phacoemulsification results and at the same time provides acceptable IOP control.

## Data Availability

The data of the current study are available from the corresponding author upon reasonable request.

## Abbreviations

UCP: Ultrasound ciliary plasty
Phaco-UCP: Combined phacoemulsification and ultrasound ciliary plasty
IOP: Intraocular pressure
AGM: Anti-glaucoma medication
MIGS: Microinvasive glaucoma surgeries
Phaco-alone: Phacoemulsification alone
Phaco-trab: Phaco-trabeculectomy
HIFU: high intensity focused ultrasound technology
Phaco-ECP: Combined Phacoemulsification and Endoscopic Cyclophotocoagulation
POAG: Primary open-angle glaucoma
LOCS III: Lens opacity classification system III
MD: Mean deviation
CDVA: Corrected Distance Visual Acuity
BSS: Balanced saline solution
SPSS: Statistical Package for Social Sciences
SD: Standard deviation
NSAIDs: Nonsteroidal Anti-Inflammatory Drugs
CME: Cystoid Macular oedema

## References

[1] Chen DZ, Koh V, Sng C, Aquino MC, Chew P. Complications and outcomes of primary phaco-trabeculectomy with mitomycin C in a multi-ethnic asian population. PLoS One. 2015;10(3):e0118852. 25775362 10.1371/journal.pone.0118852 PMC4361399

[2] Kung JS, Choi DY, Cheema AS, Singh K. Cataract surgery in the glaucoma patient. Middle East Afr J Ophthalmol. 2015;22(1):10–7. 10.4103/0974-9233.148343. 25624668 10.4103/0974-9233.148343 PMC4302462

[3] Yang HS, Lee J, Choi S. Ocular biometric parameters associated with intraocular pressure reduction after cataract surgery in normal eyes. Am J Ophthalmol. 2013;156(1):89–94.e1. 23628350 10.1016/j.ajo.2013.02.003

[4] Lin SC, Masis M, Porco TC, Pasquale LR. Predictors of intraocular pressure after phacoemulsification in primary open-angle glaucoma eyes with wide versus narrower angles (an American ophthalmological society thesis). Trans Am Ophthalmol Soc. 2017;115:T6. 29147104 PMC5665659

[5] Baek SU, Kwon S, Park IW, Suh W. Effect of phacoemulsification on intraocular pressure in healthy subjects and glaucoma patients. J Korean Med Sci. 2019;34(6):e47. 30787680 10.3346/jkms.2019.34.e47 PMC6374551

[6] O’Brien PD, Ho SL, Fitzpatrick P, Power W. Risk factors for a postoperative intraocular pressure spike after phacoemulsification. Can J Ophthalmol. 2007;42(1):51–5. 17361241 10.3129/i06-086

[7] Vass C, Menapace R. Surgical strategies in patients with combined cataract and glaucoma. Curr Opin Ophthalmol. 2004;15:61–6. 14743022 10.1097/00055735-200402000-00012

[8] Mercieca K, Shevade B, Anand N. Outcomes of combined phacoemulsification and deep sclerectomy: a 10-year UK single-Centre study. Eye (Lond). 2015;29(11):1495–503. 26337945 10.1038/eye.2015.163 PMC4815665

[9] Francis BA, Sarkisian SR, Tan JC, editors. Minimally invasive glaucoma surgery: a practical guide. 1st ed. New York: Thieme; 2017.

[10] Deb-Joardar N, Reddy KP. Application of high intensity focused ultrasound for treatment of open-angle glaucoma in Indian patients. Indian J Ophthalmol. 2018;66(4):517–23. 29582811 10.4103/ijo.IJO_1024_17 PMC5892053

[11] Melamed S, Goldenfeld M, Cotlear D, Skaat A, Moroz I. High-intensity focused ultrasound treatment in refractory glaucoma patients: results at 1 year of prospective clinical study. Eur J Ophthalmol. 2015;25(6):483–9. 25982212 10.5301/ejo.5000620

[12] Giannaccare G, Vagge A, Sebastiani S, et al. Ultrasound Cyclo-Plasty in patients with glaucoma: 1-year results from a multicentre prospective study. Ophthalmic Res. 2019;61(3):137–42. 29768281 10.1159/000487953

[13] Francis BA, Berke SJ, Dustin L, Noecker R. Endoscopic cyclophotocoagulation combined with phacoemulsification versus phacoemulsification alone in medically controlled glaucoma. J Cataract Refract Surg. 2014;40(8):1313–21. 25088629 10.1016/j.jcrs.2014.06.021

[14] Davidson JA, Chylack LT. Clinical application of the lens opacities classification system III in the performance of phacoemulsification. J Cataract Refract Surg. 2003;29:138–45. 12551681 10.1016/S0886-3350(02)01839-4

[15] Mills RP, Budenz DL, Lee PP, et al. Categorizing the stage of glaucoma from pre-diagnosis to end-stage disease. Am J Ophthalmol. 2006;141(1):24–30. 16386972 10.1016/j.ajo.2005.07.044

[16] Torky MA, Al Zafiri YA, Hagras SM, Khattab AM, Bassiouny RM, Mokbel TH. Safety and efficacy of ultrasound ciliary plasty as a primary intervention in glaucoma patients. Int J Ophthalmol. 2019;12(4):597–602. 31024813 10.18240/ijo.2019.04.12 PMC6469561

[17] Gedde SJ, Schiffman JC, Feuer WJ, Herndon LW, Brandt JD, Budenz DL, et al. Three-year follow-up of the tube versus trabeculectomy study. Am J Ophthalmol. 2009;148:670–84. 19674729 10.1016/j.ajo.2009.06.018

[18] Ruixue W, Tao W, Ning L. A comparative study between ultrasound cycloplasty and cyclocryotherapy for the treatment of neovascular glaucoma. J Ophthalmol. 2020;2020:4016536. Published 2020 Jan 22. 10.1155/2020/4016536. 10.1155/2020/4016536 PMC7001673 32047661

[19] Aptel F, Béglé A, Razavi A, et al. Short- and long-term effects on the ciliary body and the aqueous outflow pathways of high intensity focused ultrasound cyclocoagulation. Ultrasound Med Biol. 2014;40(9):2096–106. 24996576 10.1016/j.ultrasmedbio.2014.04.017

[20] Mastropasqua R, Agnifili L, Fasanella V, et al. Uveo-scleral outflow pathways after ultrasonic cyclocoagulation in refractory glaucoma: an anterior segment optical coherence tomography and in vivo confocal study. Br J Ophthalmol. 2016;100(12):1668–75. 26883868 10.1136/bjophthalmol-2015-308069

[21] De Gregorio A, Pedrotti E, Stevan G, Montali M, Morselli S. Safety and efficacy of multiple cyclocoagulation of ciliary bodies by high-intensity focused ultrasound in patients with glaucoma. Graefes Arch Clin Exp Ophthalmol. 2017;255(12):2429–35. 29043438 10.1007/s00417-017-3817-4

[22] Pellegrini M, Sebastiani S, Giannaccare G, Campos EC. Intraocular inflammation after ultrasound Cyclo Plasty for the treatment of glaucoma. Int J Ophthalmol. 2019;12(2):338–41. 30809493 10.18240/ijo.2019.02.23 PMC6376226

[23] Sun W, Yu CY, Tong JP. A review of combined phacoemulsification and endoscopic cyclophotocoagulation: efficacy and safety. Int J Ophthalmol. 2018;11(8):1396–402. 30140647 10.18240/ijo.2018.08.23 PMC6090118

[24] Pérez Bartolomé F, Rodrigues IA, Goyal S, et al. Phacoemulsification plus endoscopic cyclophotocoagulation versus phacoemulsification alone in primary open-angle glaucoma. Eur J Ophthalmol. 2018;28(2):168–74. 29077182 10.5301/ejo.5001034

[25] Hugo J, Matonti F, Beylerian M, Zanin E, Aptel F, Denis D. Safety and efficacy of high-intensity focused ultrasound in severe or refractory glaucoma [published online ahead of print, 2019 Sep 25]. Eur J Ophthalmol. 2019:1120672119874594. 10.1177/1120672119874594. 10.1177/1120672119874594 31550914

[26] Heinz C, Zurek-Imhoff B, Koch J, Rösel M, Heiligenhaus A. Long-term reduction of laser flare values after trabeculectomy but not after cyclodestructive procedures in uveitis patients. Int Ophthalmol. 2011;31(3):205–10. 21437758 10.1007/s10792-011-9440-1

[27] Tan AM, Chockalingam M, Aquino MC, Lim ZI, See JL, Chew PT. Micropulse transscleral diode laser cyclophotocoagulation in the treatment of refractory glaucoma. Clin Experiment Ophthalmol. 2010;38(3):266–72. 20447122 10.1111/j.1442-9071.2010.02238.x

[28] Aquino MC, Barton K, Tan AM, et al. Micropulse versus continuous wave transscleral diode cyclophotocoagulation in refractory glaucoma: a randomized exploratory study. Clin Experiment Ophthalmol. 2015;43(1):40–6. 24811050 10.1111/ceo.12360

[29] Liu GJ, Mizukawa A, Okisaka S. Mechanism of intraocular pressure decrease after contact transscleral continuous-wave Nd:YAG laser cyclophotocoagulation. Ophthalmic Res. 1994;26(2):65–79. 8196935 10.1159/000267395

[30] Sousa DC, Ferreira NP, Marques-Neves C, et al. High-intensity focused ultrasound Cycloplasty: analysis of pupil dynamics. J Curr Glaucoma Pract. 2018;12(3):102–6. 31354201 10.5005/jp-journals-10078-1232 PMC6647825

[31] Rivero-Santana A, Pérez-Silguero D, Pérez-Silguero MA, Encinas-Pisa P. Pupil Ovalization and accommodation loss after high-intensity focused ultrasound treatment for glaucoma: a case report. J Curr Glaucoma Pract. 2019;13(2):77–8. 31564798 10.5005/jp-journals-10078-1256 PMC6743310

[32] Bolek B, Wylegala A, Mazur R, Wylegala E. Pupil irregularity after ultrasound ciliary plasty in glaucoma treatment. Acta Ophthalmol. 2019;97(S263); Special Issue: Abstracts from the 2019 European Association for Vision and Eye Research Conference (December 2019). 10.1111/j.1755-3768.2019.5480.

[33] Yip LW, Yong SO, Earnest A, Ji J, Lim BA. Endoscopic cyclophotocoagulation for the treatment of glaucoma: an Asian experience. Clin Experiment Ophthalmol. 2009;37(7):692–7. 19788666 10.1111/j.1442-9071.2009.02120.x

