# Editorial Expression of Concern: Phaco-UCP; combined phacoemulsification and ultrasound ciliary plasty versus phacoemulsification alone for management of coexisting cataract and open angle glaucoma: a randomized clinical trial

**DOI:** 10.1186/s12886-024-03582-8

**Published:** 2024-07-30

**Authors:** Magda A. Torky, Yousef A. Alzafiri, Ameera G. Abdelhameed, Eman A. Awad

**Affiliations:** 1 Department of Ophthalmology, Mansoura Ophthalmic Center, Faculty of medicine, Mansoura University, Al-Gomhoria Street, Mansoura, 35516 Egypt; 2 Department of Ophthalmology, Dar Al Shifa hospital, Hawally City, 30000 Kuwait

The Editor of the journal would like to alert the readers that concerns regarding consent to participate from study participants were raised for this article [1]. Despite the efforts made to obtain an explanation and clarification from the authors regarding this concern, it appeared that no documentary evidence could be made available by the authors to support their explanation.

All authors disagree with this Editorial Express of Concern.

## References

[1] Torky, M.A., Alzafiri, Y.A., Abdelhameed, A.G. et al. Phaco-UCP; combined phacoemulsification and ultrasound ciliary plasty versus phacoemulsification alone for management of coexisting cataract and open angle glaucoma: a randomized clinical trial. BMC Ophthalmol 21, 53 (2021). 10.1186/s12886-021-01818-5 10.1186/s12886-021-01818-5 PMC7819220 33478426

